# Molecular, Cellular and Functional Analysis of TRγ Chain along the European Sea Bass *Dicentrarchus labrax* Development

**DOI:** 10.3390/ijms22073376

**Published:** 2021-03-25

**Authors:** Andrea Miccoli, Laura Guerra, Valeria Pianese, Paolo Roberto Saraceni, Francesco Buonocore, Anna Rita Taddei, Ana Couto, Tania De Wolf, Anna Maria Fausto, Giuseppe Scapigliati, Simona Picchietti

**Affiliations:** 1 Department for Innovation in Biological, Agro-Food and Forest Systems, University of Tuscia, Largo dell’Università, 01100 Viterbo, Italy; andrea.miccoli@unitus.it (A.M.); lauraguerra@unitus.it (L.G.); vale.p2295@gmail.com (V.P.); paoloroberto33@gmail.com (P.R.S.); fbuono@unitus.it (F.B.); fausto@unitus.it (A.M.F.); scapigg@unitus.it (G.S.); 2 Section of Electron Microscopy, Great Equipment Center, University of Tuscia, 01100 Viterbo, Italy; artaddei@unitus.it; 3 Interdisciplinary Centre of Marine and Environmental Research (CIIMAR), University of Porto, Av. General Norton de Matos, 4450-208 Matosinhos, Portugal; acouto@fc.up.pt; 4 INVE Aquaculture Research Center, 57016 Rosignano Solvay, Italy; t.dewolf@inveaquaculture.com

**Keywords:** T cell receptor chain gamma, immune system, ontogeny, intestine, nutritional immunity

## Abstract

In jawed vertebrates, adaptive immune responses are enabled by T cells. Two lineages were characterized based on their T cell receptor (TcR) heterodimers, namely αβ or γδ peptide chains, which display an Ig domain-type sequence that is somatically rearranged. γδ T cells have been less extensively characterized than αβ and teleost fish, in particular, suffer from a severe scarcity of data. In this paper, we worked on the well-known model, the European sea bass *Dicentrarchus labrax,* to broaden the understanding of teleost γδ-T cells. The T cell receptor chain (TR) γ transcript was expressed at a later developmental stage than TRβ, suggesting a layered appearance of fish immune cells, and the thymus displayed statistically-significant higher mRNA levels than any other organ or lymphoid tissue investigated. The polyclonal antibody developed against the TRγ allowed the localization of TRγ-expressing cells in lymphoid organs along the ontogeny. Cell positivity was investigated through flow cytometry and the highest percentage was found in peripheral blood leukocytes, followed by thymus, gut, gills, spleen and head kidney. Numerous TRγ-expressing cells were localized in the gut mucosa, and the immunogold labelling revealed ultrastructural features that are typical of T cells. At last, microalgae-based diet formulations significantly modulated the abundance of TRγ^+^ cells in the posterior intestine, hinting at a putative involvement in nutritional immunity. From a comparative immunological perspective, our results contribute to the comprehension of the diversity and functionalities of γδ T cells during the development of a commercially relevant marine teleost model.

## 1. Introduction

T cells are generally considered the master players that enable the adaptive immune responses in jawed vertebrates. In the classical view, they recognize a major histocompatibility complex (MHC)-presented or naïve antigen through their CD3-associated surface antigen receptors. Non-self recognition is performed by two types of T cell lineages identified according to their TcR heterodimers (αβ or γδ peptide chains) [1].

While all TcRs, independently of their polypeptide chains, share an Ig domain-type sequence composed of multiple segments that undergo somatic rearrangement and result in an extremely diversified repertoire, several distinctions were evidenced comparing αβ and γδTcRs.

Firstly, a more in-depth knowledge exists about the former: α and β chains were identified in all vertebrate classes, e.g., birds [2], amphibians [3], reptiles [4], bony fish [5] and elasmobranchs [6], and were among the first cellular immune receptors to be cloned in fish [7]. Whereas the αβTcR complex was elucidated in terms of molecular structure, genetics and antigen-recognition processes in several fish species, the existence of a human γδTcR homologue was reported by *in silico* prediction for the first time in the Japanese flounder *Paralichthys olivaceus* [8]. Teleost-wise, *TRγ* and/or *TRδ* genes were then described only in the common carp *Cyprinus carpio* [9], Atlantic salmon *Salmo salar* [10], European sea bass *Dicentrarchus labrax* [11], channel catfish *Ictalurus punctatus* [12], mandarin fish *Siniperca chuatsi* [13], zebrafish *Danio rerio* [14] and dojo loach *Misgurnus anguillicaudatus* [15]. Through the characterization of CDR3 loops, γδTcR appears more similar to Ig than it is to αβTcR [8].

Secondly, T cell populations differ according to their anatomical locations: αβTcR receptor is mostly expressed by circulating T cells, whereas γδTcR is principally localized in mucosal tissues such as skin, gills and intestine. The relevance of γδ T cells in such tissues can be argued from the spontaneous RAG1-driven somatic recombination of V-D-J segments in fish gut [11]. Basal TRγ expression was qualitatively defined in the common carp [9], Atlantic salmon [10], European sea bass [16], olive flounder [17] and mandarin fish [13]. In carp, higher transcription was found in gills than in thymus [9], which is the site where the majority of αβTcR-bearing T cells mature [18]. However, an inter-species comparison was not allowed because such analyses were performed on fish of different age, and both the expression and function of TRγ are known to be age-dependent [9]. Additionally, the transcript was not actually quantified, either relatively or absolutely.

Thirdly, by summarizing information retrieved from mammals and teleost models, γδ T cells have distinct peculiar features, in fact: they (i) are not MHC-restricted; (ii) have natural killer (NK)-cell reactivities and kill stressed, infected and tumoral cells; (iii) have a CD4^−^ CD8^+^ phenotype and show phagocytic activity against soluble and particulate antigens; (iv) recognize unconventional antigens including phosphorylated molecules, metabolic intermediates and lipids; (v) may act as pattern recognition receptors and participate in the humoral adaptive response by initiating CD4^+^ T cell proliferation, inducing B cell activation and IgM production; and (vi) have a role in the production of the mucosal immunity-related fish-specific IgT/Igz isotype [12,14,19,20,21]. Based on these findings, mammalian γδ T cells were proposed as a primordial lymphocyte population predating αβ T cells and B cells and accordingly located at the interface between innate and acquired immunities [22]. Recent evidences show that fish lymphocyte subpopulations behave as innate-like mammalian cells, according to the review of Scapigliati et al. [23].

In this paper, building on the work by Buonocore et al. [11], we leveraged on molecular, biochemical and immuno-related techniques with the aim of broadening the understanding of teleost TRγ along the ontogeny and at the juvenile stage of the European sea bass *Dicentrarchus labrax*. A severe scarcity of data afflicts teleost immune ontogeny: only one study addressed the transcriptional pattern of TRγ up to 35 days post hatching (dph) [17], and the immunohistochemical localization was never investigated. *D. labrax* is an important marine model species for immunobiological studies due to the availability of tools that have allowed the elucidation of specific physiological features of T lymphocytes from an evolutionary perspective. This knowledge, in turn, has improved sea bass farming with positive commercial consequences. In this regard, microalgae have received a growing interest in aquaculture as a method for increasing the industry sustainability, because they contain several functional compounds characterized by immunostimulatory, antioxidant or anti-inflammatory activities [24,25,26,27,28]. Based on a recent work that tested the marine microalgae *Nannochloropsis* sp. effects on sea bass intestinal immunity [29], we herein assessed the outcomes on intestinal γδ T cells.

Taken together, we expect our results to increase the comprehension of the diversity and functionalities of γδ T cells in teleosts, in view of a possible interdisciplinary exploitation as per cutting-edge research [30].

## 2. Results

### 2.1. Antibody Production, ELISA Validation and IgG Enrichment

To obtain a marker of TRγ^+^ cells, two antisera were produced by immunizing two New Zealand rabbits. The antibodies were named RaTRγ1 and RaTRγ2 and tested in an ELISA assay. The antiserum RaTRγ1 was selected based on its greater performance evidenced by ELISA assay results (Figure S1) and purified with Protein G Sepharose^®^ (Abcam, catalog #ab193259) for IgG enrichment. The obtained fraction was named RaTRγ1 IgG and used for all experiments herein reported.

### 2.2. Immunoprecipitation

**Figure 1:**
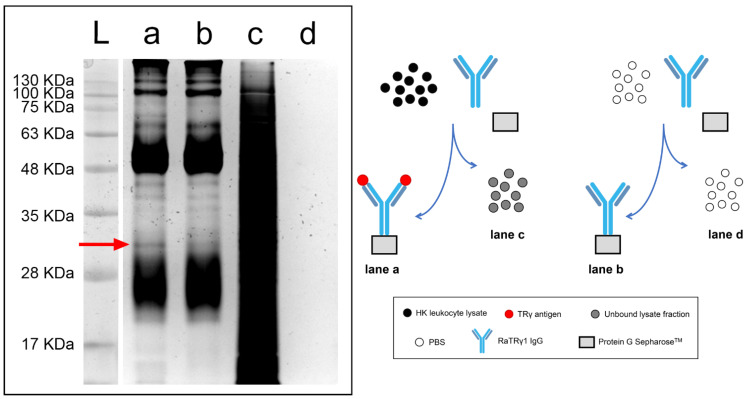
Immunoprecipitation analysis. SDS-PAGE of HK leukocyte lysate immunoprecipitated with RaTRγ1 IgG and stained with the ProteoSilver™ Stain kit. Lane a: Protein G Sepharose^®^-precipitated PAb incubated with the HK leukocyte lysate. Lane b: Protein G Sepharose^®^-precipitated PAb incubated with PBS. Lanes c and d: PAb-unbound supernatant fraction of HK leukocytes and PBS, respectively. The red arrow indicates the TRγ antigen, which is specifically recognized by RaTRγ1 IgG. The ladder is indicated by L. On the right, a schematic representation of the SDS-PAGE results.

To verify the specificity of RaTRγ1 IgG, an immunoprecipitation experiment was performed. The silver-stained electrophoretic profile of head kidney (HK) leukocytes immunoprecipitated with RaTRγ1 IgG is shown in Figure 1. Lanes a and b, in which either the HK leukocyte lysate or PBS, respectively, had been incubated with RaTRγ1 IgG and Protein G Sepharose^®^, differed by a band of approximately 33 kDa MW which was evident only in lane a (red arrow). The size of 33 kDa was the one expected by sea bass TRγ AA sequence without the signal peptide (accession # FR745889.1), confirming that PAb RaTRγ1 IgG specifically and uniquely binds the TRγ antigen. The amount of protein starting material was abundant, as evidenced by the unbound supernatant fraction of the HK leukocyte lysate (lane c). Instead, the supernatant resulting from the incubation of PBS with RaTRγ1 IgG and Protein G Sepharose^®^ did not contain any protein band (lane d) and was free of any Protein G Sepharose^®^ contamination.

### 2.3. Expression Analysis of T Cell Markers along the Ontogeny

**Figure 2:**
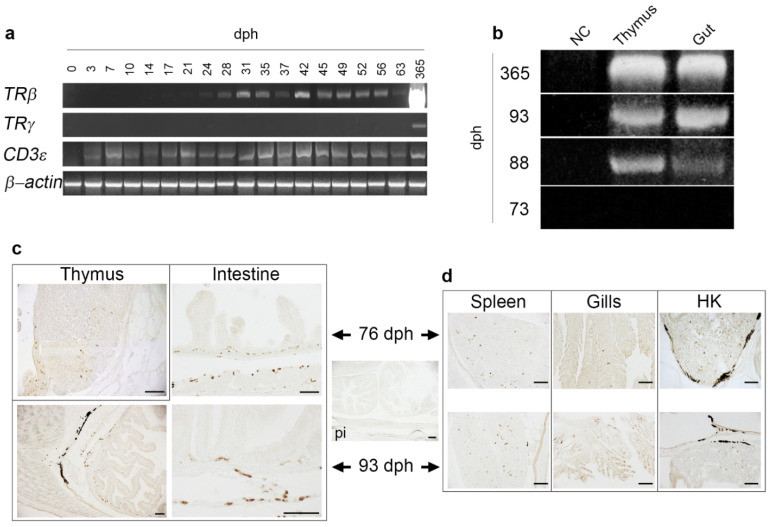
(**a**) RT-PCR of different T cell markers along the first 63 days of *D. labrax* ontogeny and in a thymus of a 365-day-old specimen. *β-actin* served as housekeeping gene. (**b**) RT-PCR of *TRγ* performed on two immune-relevant tissues at 73–93 dph ontogeny and 365-day-old stages. (**c**,**d**) Immunohistochemistry photographs of thymus, intestine, spleen, gills and HK of 76- and 93-dph larvae stained with RaTRγ1 IgG diluted 1:100 in PBS. Scale bars are all 50 μm.

To investigate the sea bass temporal mRNA expression of some T cell markers, PCR analysis was performed. The expression of *TRγ*, *TRβ* and *CD3ε* along developmental stages and 1-year-old (365 dph) specimens is shown in Figure 2a,b. The absence of DNA contamination and the comparable amounts of mRNA in the samples used for the RT-PCR analysis were monitored using specific primers for the housekeeping gene *β-actin* that bracketed an intron. The expression of *TRγ* was undetected from hatching to 73 dph (Figure 2a). Later, between 74 and 88 dph, *TRγ* expression appeared both in thymus and intestine (Figure 2b). The day 88 amplicon, which was cloned and sequenced, confirmed the sea bass *TRγ* identity. Thymic (Figure 2a,b) and intestinal (Figure 2b) expression of *γ* chain increased in 1-year-old sea bass. On the other hand, mRNA expression of *TRβ* was detected at earlier developmental stages and first appeared between 21 and 24 dph, and increased along the development (Figure 2a). *TRβ* transcripts detected in thymus of 1-year-old specimens were regarded as positive controls (Figure 2a). At last, first expression of *CD3ε* was detected at day 3 ph and likely appeared between 1 and 3 dph; the transcripts visually appeared to increase along the development until 63 dph (Figure 2a). The day 0, 3, 7, 14, 21 and 63 amplicons were cloned and sequenced, confirming sea bass *CD3ε* identity.

### 2.4. Localization of TRγ^+^ Cells in Developing Lymphoid Organs and Mucosal Tissues of Post-Larvae

Immunohistochemistry (IHC) of sea bass post-larvae at 76 and 93 dph allowed the localization of TRγ^+^ cells in developing lymphoid organs and mucosal tissues (Figure 2c,d). At 76 dph, rare TRγ^+^ cells were found in the thymic medulla and in the pharyngeal epithelium covering the gland. At this stage, TRγ^+^ cells are housed in the lamina propria (LP) of intestinal segments. At 93 dph the abundance of TRγ^+^ cells increased, although the stained cells never formed lymphoid aggregates in the LP. Pre-immune serum (PI) did not show any reactivity. Isolated and infrequent TRγ^+^ cells were also localized in the spleen, secondary lamellae of gills and HK both at 76 and 93 dph. The abundance of positive cells increased at this latter stage.

### 2.5. Transcriptional and Translational Quantification of TRγ in Lymphoid Organs and Mucosal Tissues of One-Year-Old Fish

**Figure 3:**
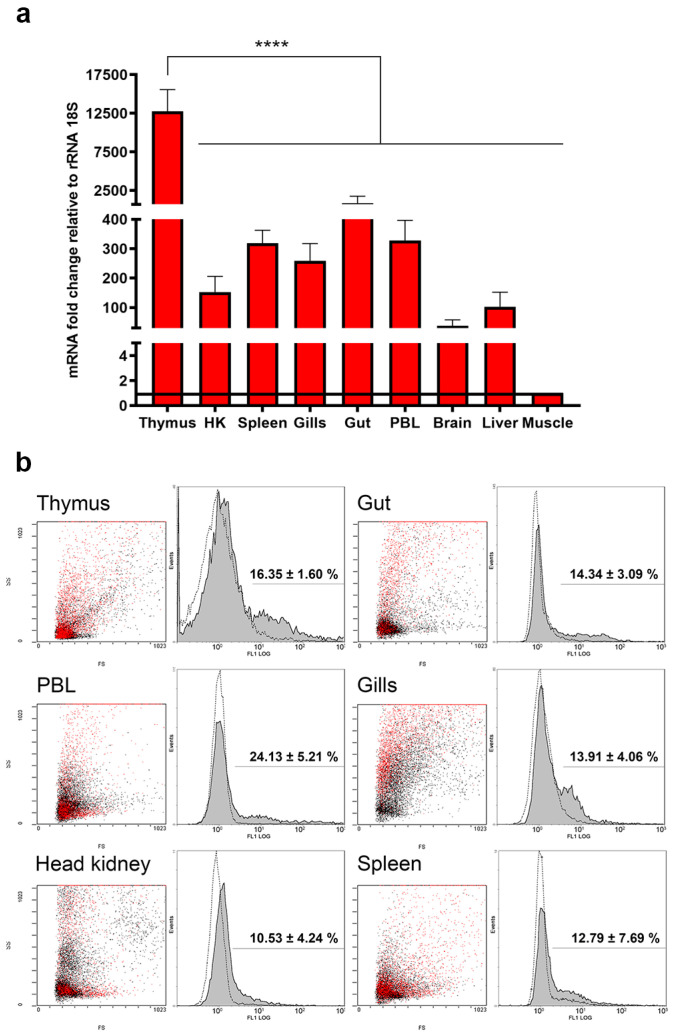
(**a**) Real-time qPCR of *TRγ* basal transcription levels in 1-year-old fish. mRNA fold change is expressed relatively to *rRNA 18S* as housekeeping gene. The muscle tissue was used as calibrator (i.e., tissue with the lowest relative expression of 1, highlighted by the straight horizontal line). Data is presented as mean + SD (*n* = 3). **** : *p* < 0.0001. (**b**) Flow cytometry of leukocytes from 1-year-old fish incubated with RaTRγ1 IgG at a 1:20 dilution in PBS. Red-colored events in the FS/SS dot-plots (left for each tissue) correspond to gated events in the FL1 histograms (right). Positivity percentage is presented as mean ± SEM (*n* = 3) (gray peaks, solid line) net of pre-immune positivity (white peaks, dashed line).

The constitutive expression of *TRγ* mRNA was investigated by real-time qPCR in healthy sea bass (Figure 3a). In one-year-old specimens, the transcript had the highest abundances in lymphoid organs (i.e., thymus and spleen) and mucosal tissues (i.e., gut and gills). The expression in spleen was similar to that in peripheral blood lymphocytes (PBL). There was a significant effect of the examined tissues on mRNA expression (*F*_8,18_ = 53.37, *p* < 0.0001) and only the transcriptional differences between thymus and any other tissue were significant (adjusted *p* value < 0.0001).

TRγ immunophenotyping was achieved by flow cytometry analysis of PFA-fixed cells incubated with PAb RaTRγ1 IgG (Figure 3b). The quantitative assessment revealed that the highest percentage of TRγ^+^ cells was found in PBL (24.13 ± 5.21%), followed by thymus (16.35 ± 1.6%) and gut (14.34 ± 3.09%). Gills and spleen returned similar values (13.91 ± 4.06% and 12.79 ± 7.69%, respectively) while positivity in HK amounted to 10.53 ± 4.24%. For each tissue, gated fluorescent events in FL1 histograms were highlighted in red in FS/SS dot-plots and appeared to possess the typical features of lymphocyte populations, size- and granularity-wise.

### 2.6. Localization of TRγ^+^ Cells in Lymphoid Organs and Mucosal Tissues of One-Year-Old Fish

IHC, indirect immunofluorescence (IIF) and immunogold analyses were employed to provide the most detailed localization and ultrastructural features of RaTRγ1 IgG-marked cells. 

**Figure 4:**
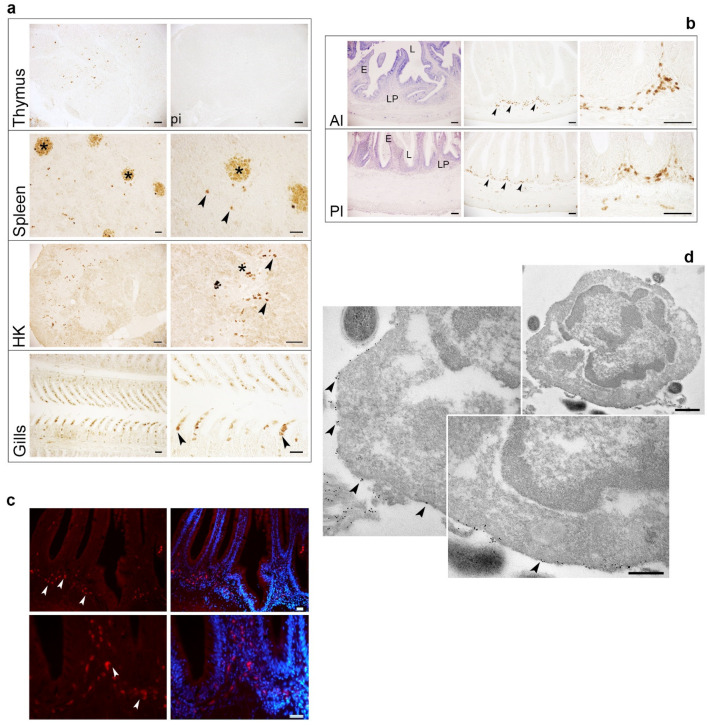
Localization of TRγ^+^ cells in lymphoid organs and mucosal tissues of one-year-old fish. (**a**,**b**) Immunohistochemistry photographs of thymus (scale bars: 50 μm), spleen (scale bars: 20 μm), HK (scale bars: 50 μm), gills (scale bars: 20 μm) and intestine (scale bars: 50 μm) of one-year-old fish. Asterisks in spleen and HK photographs indicate MMCs. LP, E and L in intestine photographs indicate lamina propria, epithelium and lumen, respectively. Black arrowheads are examples of TRγ^+^ cells. (**c**) Indirect immunofluorescence analysis on posterior intestine sections. Examples of immunoreactive cells are indicated by white arrowheads. Scale bars are all 20 μm. (**d**) Pre-embedding immunogold labelling of cell surface TRγ. Examples of electron-dense gold nanoparticles are indicated by black arrowheads. Scale bars are 500 and 250 nm for the lower and greater magnification photographs, respectively.

IHC analysis of lymphoid organs and mucosal tissues is shown in Figure 4a. Isolated TRγ^+^ cells were found in the thymic cortico-medullary region and in the medulla. The staining was not detected in sections probed with the pre-immune serum (PI). Rare and isolated positive cells were distributed throughout the splenic parenchyma, often in close proximity to melano-macrophage centres (MMCs). Differently, the HK housed TRγ^+^ cells mainly located in the lymphoid areas, usually endowed with MMCs. HK myeloid areas did not exhibit T cells expressing the TRγ. A moderate abundance of TRγ^+^ cells was also observed within the secondary lamellae of gills.

The histology and IHC of the anterior intestine (AI) and posterior intestine (PI) segments in healthy sea bass individuals are shown in Figure 4b. IHC analysis performed on these specimens allowed the detection of TRγ^+^ cells in intestinal regions. In particular, the LP of both AI and PI segments housed numerous strongly stained cells. Rare TRγ^+^ cells, displaying a faint staining intensity, were found in the epithelium of the AI segment, whereas isolated stained cells were occasionally observed among the enterocytes in the PI segment.

IIF confirmed the prevalence of TRγ^+^ cells in the sea bass gut (Figure 4c). Intestinal T cells bearing the TRγ considerably accumulated in the LP of the PI segment. The morphology and the membrane staining of the immunoreactive cells can be appreciated from the corresponding higher magnification photographs.

Pre-embedding immunogold labelling of isolated intestinal leukocytes revealed the fine localization of the target protein and defined the ultrastructural features of the recognized cells (Figure 4d). Numerous gold nanoparticles were exclusively distributed on the plasma membrane of intestinal leukocytes that had the typical features of T cells: a large and heterochromatic nucleus and a thin layer of cytoplasm. The nanoparticle distribution appeared clustered on the T cell plasma membrane.

### 2.7. Effects of Marine Microalgae Nannochloropsis sp. Diets on Intestinal TRγ-Expressing Cells

The effect of diet on intestinal T cells expressing the TRγ was evaluated in sea bass fed with fish meal and fish oil increasingly replaced by the marine microalgae *Nannochloropsis* sp.

**Figure 5:**
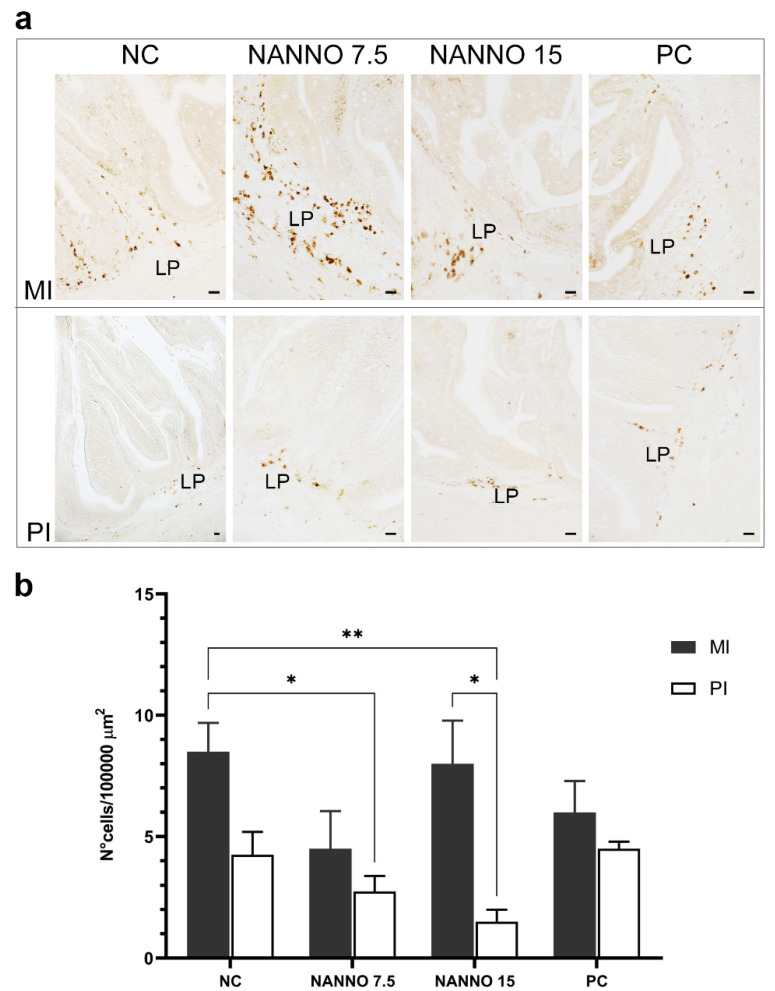
(**a**) Immunohistochemistry photographs of MI and PI following the dietary inclusion of *Nannochloropsis* sp. IHC experimental conditions are as above. LP indicates lamina propria. Scale bars are all 20 μm. (**b**) TRγ^+^ cells density over a 100,000 μm^2^-area following dietary exposure to *Nannochloropsis* sp. * = *p* < 0.05; ** = *p* < 0.01. NC = negative control diet; NANNO 7.5 and 15 = diet formulations with 50 and 100% fish meal replacement by *Nannochloropsis* sp. biomass, respectively; PC = positive control diet.

The localization and abundance of TRγ^+^ cells were analyzed in the mid intestine (MI) and PI segments (Figure 5a). In all experimental groups, T cells expressing the γ chain were rarely located in the intestinal epithelium, while they were abundantly detected in the LP of both MI and PI segments. A significantly lower density of TRγ^+^ cells was found in the mucosa of the PI segment of fish fed with NANNO 7.5 and NANNO 15 compared to the MI segment of the negative control (NC) group (Figure 5b). Within the experimental group NANNO 15, a significant decrease in TRγ^+^ cells was found in the PI compared to the MI. The density of TRγ^+^ cells was not significantly affected in either the MI or PI segments of both NC and positive control (PC) groups.

## 3. Discussion

The T cells of all jawed vertebrates play pivotal functions in the adaptive immune system [31,32], recognizing pathogens and discriminating between self and non-self antigens by using somatically diversified TcRs. Four TRs (α, β, γ and δ) were identified [33], and the genes encoding for these subunits were demonstrated to be highly conserved both in sequence and organization along gnathostome evolution [31,32]. As an exception, the genome of nonplacental mammals (monotremes and marsupials) encode for a fifth TR, namely TRμ, which undergoes somatic diversification [34,35,36]. Based on the composition of their TR heterodimers, T cells can be divided into two distinct lineages: αβ T cells and γδ T cells [37,38]. The functional delineations between αβ- and γδ-T subsets have not yet been clarified in jawed vertebrates, but many unique features of γδ T lymphocytes have emerged in mammals [39]. A broad range of immune responses including tumour surveillance, innate responses to pathogens and stress, wound healing, renewal of barrier tissues, surveillance and shaping of adipose tissue were associated with the major lineages of γδ T cells in mice and humans [39,40,41,42,43]. γδ T cells are still an enigmatic subset in teleost fish: to our knowledge, only one study has explored their cell biology in fish, providing evidence of the functions played in adaptive humoral immunity [14]. Three innovative aspects can be recognized in our study: (i) the first description of the *TRγ* expression in the developing European sea bass up to 93 dph; (ii) the localization of TRγ-expressing cells in lymphoid organs and mucosal tissues, which was allowed by the production of a specific antibody; and (iii) the involvement of γδ T cells in response to alternative microalgae-based diets.

When they hatch, teleost fish have a much less developed adaptive immune system than eutherian mammals at birth. During the critical period that follows, when the endogenous adaptive immune system remains undeveloped, newborn fish depend on their innate immune system for survival [44,45]. To better understand the development of immunocompetence and identify the earliest T cell development in sea bass, we focused on the first appearance of mature TRγ, TRβ and CD3ε transcripts. The results we obtained were unexpected both in terms of individual and collective transcriptional timing: mature CD3ε transcripts were detected already at 3 dph, when the lymphoid tissue is still undifferentiated, whereas TRβ expression was found relatively early along sea bass development (between day 21 and 24 ph), when the thymus is still absent [46]. In previous reports, sea bass paired thymic anlage first acquired a lymphoid appearance at 27 dph [46], and only three days later were T cells detected in the gland using the pan-T cells MAb DLT15 [47]. At this age, the sea bass thymus lacks defined corticomedullary demarcation. Taking into consideration that the thymic regionalization is defined from 51 dph and that the histology of the gland is well established in three-month-old fish, it is implausible that the early expression of *CD3ε* or *TRβ* indicates the presence of mature and functional T cells; rather, they may reveal the occurrence of cells committed to the T cell lineage (thymocyte progeny). In teleost fish the mechanisms behind lineage fate decision of the cells is yet unknown. Nevertheless, we cannot rule out that early *CD3ε* and *TRβ* expressed by the thymocyte progeny contribute to thymus differentiation or instead represent sterile/aberrant transcripts, which are reported to be abundant in teleosts [48]. Interestingly, *TRγ* expression was detectable along T cell ontogeny much later than *TRβ* and when a well-defined thymic regionalization was observed in sea bass [47,49,50]. This result is in contrast to placental mammals and birds, where the development of γδ T cells generally precedes that of αβ T cells [20,51], but is consistent with the situation in the marsupial *Monodelphis domestica*, where mature *TRα* and *TRβ* transcripts are detected earlier than *TRγ* transcripts, and *TRγ* transcripts occur concomitantly with the appearance of a distinct thymic cortical region [52]. This suggests that αβ T cells could develop before γδ T cells. The transcriptional timing of TR subunits was also described in early stages of the teleost *Paralichthys olivaceus*: TRα was detected prior to hatching but *TRβ* and *TRγ* or *TRδ* appeared to be transcribed at 1 and 0 dph, respectively [17]. It is possible that TR subunits have a functionally diverse relevance according to the time of expression, as suggested by the authors. Moreover, the production of diverse types of lymphocytes and the diverse timing of αβ and γδ T cell lineages may imply a “layered” production of fish immune cells.

In this paper, an antibody specific for sea bass TRγ was developed and exclusively recognized an expected band of approximately 33 kDa (Figure 1, red arrow), as per the silver-stained profile of immunoprecipitated HK leukocytes. The antibody, for the first time in a teleost fish, has allowed the localization of the γ-chain-expressing T cells along the ontogeny. TRγ was expressed in the thymus, HK, spleen, gills and intestine of sea bass post-larvae at 76 dph. From this stage to 1-year-old specimens, TRγ^+^ cells were mainly localized in the thymic corticomedullary region. At this age the thymus, as previously demonstrated, housed TRβ^+^ and CD8α^+^ CD4^+^ double positive (DP) cells mainly localized in the cortex, and CD8α^+^ or CD4^+^ single positive cells in the medulla, as a result of αβ T lineage maturation, which depends on αβ TR-mediated positive and negative selection [49,50]. The unique localization of sea bass TRγ^+^ cells seems to be consistent with the well-known notion that mammal γδ T cells, unlike αβ T cells, do not require clonal expansion or differentiation from a naïve cell for their effector responses [53], and develop following a single γδ selection step mediated by the γδ TR [54]. In mammals, double negative (DN) thymocytes undergo γδ selection and become immature γδ thymocytes [51]. These cells have been demonstrated not to progress through a DP stage [55]; however, the lymphotoxin production by DP αβ thymocytes has been identified as the mechanism regulating γδ T cell effector fate acquisition, thereby facilitating fast responsiveness [51,56,57,58]. Taking this aspect into consideration, we formulate the hypothesis that in sea bass, similarly to what was reported in mammals [51], resident developing thymocytes (TRβ-, CD4- and CD8α-expressing cells) [49,50] and non-lymphoid thymic stromal cells [59], detectable in the regionalized thymus before the appearance of TRγ cells, may play important roles in driving γδ T innate-like early responders. However, it cannot be ruled out that some γδ T cells could exit the thymus as naïve cells and acquire effector function following activation in the periphery, as reported for mammals [54]. These cells are referred to as “inducible” γδ T cells [54]. Interestingly, spectratyping of sea bass TRγ CDR3 regions revealed different lengths between the thymus and gut of the same animal for a given V/C combination, suggesting that diverse repertoires are expressed in these tissues [11].

In one-year-old specimens, TRγ^+^ cells are a distinct lineage and constitute 16.35 ± 1.6% of the total thymic T cell pool. From the thymus these cells take up residency in a variety of tissues to perform activities common to both innate and adaptive immunity, as demonstrated in mammals and zebrafish [14,39,60]. These unconventional T cells yielded the highest proportion in sea bass peripheral blood followed by thymus, mucosal tissues (intestine and gills), spleen and HK, and overall accounted for 10.53–24.13% of total lymphocytes, with a similar proportion seen in zebrafish peripheral blood, HK and spleen [14]. Instead, qPCR analysis revealed the highest abundance of *TRγ* transcripts in the thymus, whose expression exceeded that of any other tissue, as previously reported [16]. Taking for granted that transcripts and protein levels do not always vary accordingly, such an apparent divergence may be tentatively explained by the fact that fish thymocytes require greater transcriptional rates than peripheral/circulating lymphocytes for T lineage decision. In mammals, thymic T lineage decision and effector function acquisition are two sequential steps in γδ T cells’ commitment that were tentatively explained by the signal strength model and the stochastic-selective (pre-commitment) model [55]. The signal strength model links the TR of DN thymocytes to numerous environmental factors [61], with weak or strong signals promoting αβ or γδ fates, respectively [51].

In mammals, distinct barrier tissues endowed with non-redundant functions house highly specialized γδ T cell subsets that are not found elsewhere in the body [39,51]. These naturally tissue-resident γδ T cells are highly adapted lymphocytes that actively survey neighbouring cells, sense and respond to stresses of various nature and participate in many tissue processes [62]. The sea bass intestinal mucosa is armed with numerous T cells [63,64], a large fraction of which express TRβ and CD8α transcripts [65]. Moreover, DLT15-immunopurified intestinal T cells demonstrated a very high expression of TRγ [16]. Herein, we provide further insights into sea bass intestinal resident T cells, demonstrating that gut mucosa is colonized by TRγ-expressing cells. Through the immunogold labelling we evidenced that this distinct T cell lineage possesses typical lymphocyte morphology, with round dense nuclei surrounded by a thin round ring of cytoplasm, and is randomly distributed on the plasma membrane, probably for an optimized fast recognition of antigens. Future efforts will provide a spatial distribution of TRγ on the plasma membrane and a quantitative estimation of the degree of randomness.

Zebrafish and mammalian γδ T cells exhibit the surface CD4^−^ CD8^+^ phenotype [14]. Specific antibodies for CD8 or CD4 T cell subsets are not yet available for sea bass, however, in situ hybridization analysis revealed the presence of rare CD4- and numerous CD8α-expressing cells in the sea bass intestinal mucosa, with CD8α cells being mainly localized in the LP [65]. In this paper we demonstrated that such an intestinal compartment is colonized by abundant TRγ^+^ cells, as reported in zebrafish [14]. The unique localization of these unconventional cells differs from that found in mammals, where a gradient with an increasing proportion of TRγ^+^δ^+^ CD8αα^+^ cells was found towards the gut lumen, with the highest number detected within the epithelium [66]. Moreover, it is well known that mammalian gut lymphocytes express various receptors and cytokines according to their location. In particular, resident LP TRγ^+^δ^+^ CD8^+^ cells were demonstrated to produce IL-10 and IL-17 and display a memory-like phenotype [54,66]. In sea bass, the intestine was found to express the highest level of IL-10 [16]; however, further studies will be necessary to better characterize the phenotypes and the activation patterns of the γδ T cells and in particular the roles they play in immunosurveillance.

Although feeding habits and the intestinal microbiome are known to modulate the numerosity of T cells in salmon [67] and sea bass [68], and in general interact with the host immune system [27], limited research has demonstrated that nutrients and bioactive food components are common dietary modifiers of vertebrate γδ T cells in terms of abundance, cytotoxicity, cytokine secretion and proliferative capacity [69]. For a better comprehension of the biology of these resident T cells, we evaluated whether an alternative diet based on the marine microalgae *Nannochloropsis* sp. could modulate sea bass intestinal γδ T cells. It must be highlighted that recent data demonstrated that the same microalgae species is able to modulate CD3ε^+^ T lymphocytes in sea bass [29]. Herein, dietary microalgae incorporation effects on the gut immune system induced a significant decrease in mucosal TRγ^+^ cells in the posterior intestine of fish fed with the NANNO 7.5 and NANNO 15 diets. This intestinal segment has a fundamental role in active immunosurveillance [70] and is enriched with T lymphocytes [63,64] and CD8α-expressing cells [65]. Interestingly, a physiological impact on LP-resident TRγ cells was demonstrated, further confirming the existence of a diet–γδ T cells relationship whose physiological significance is not simple to clarify. A similar outcome was witnessed at the mRNA level following the in vivo infection of sea bass with a retrovirus (*Betanodavirus*) [11]. Additional research needs to be performed to clarify the biological significance of transcript down-regulation and/or the redistribution of γδ T cells. In fact, the lack of enhancement of immune functions does not necessarily translate to decreased health, e.g., over-activated γδ T cells may enhance the pathology associated with inflammatory bowel [71] or celiac disease in humans [72].

In conclusion, from a comparative immunological perspective, the present paper provides novel information of some physiological features associated with TRγ-expressing cells along the development of a commercially relevant marine teleost species, and suggests a possible relationship between nutritional immunity and intestinal γδ T cells.

## 4. Materials and Methods

### 4.1. Animal Husbandry and Sampling

**Figure 6:**
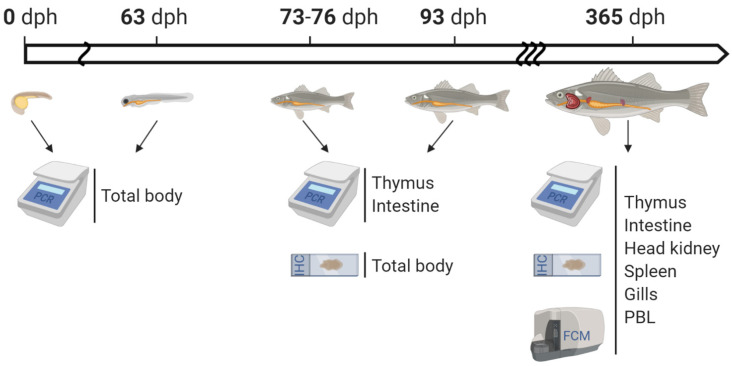
Diagram of the experimental design including ontogeny and juvenile stage details and experimental techniques used. PCR = polymerase chain reaction; IHC = immunohistochemistry; FCM = flow cytometry. PBL was analyzed by PCR and FCM. The illustration was created with BioRender.com.

All analyses described in the following sections were performed on European sea bass *Dicentrarchus labrax* L. (Figure 6). The ontogeny investigations at both the transcript and protein level were performed on larvae and post-larvae of up to 93 dph from the INVE Aquaculture Research Center in Rosignano Solvay (Italy). Healthy 1-year-old specimens of variable sizes were instead obtained from a commercial aquaculture facility in Orbetello (Italy). Organs and tissues (spleen, PBL, brain, liver, anterior intestine, mid intestine, posterior intestine, thymus, HK, gills and muscle) were dissected following lethal anesthesia and used accordingly, as described below. The diet trials took place at CIIMAR experimental facilities using fish from the commercial hatchery in Acuinuga (Spain).

In all cases, fish were anesthetized with 0.3 ml L^−1^ of ethyleneglycol monophenyl ether (Merck, catalog #8072911000) and all efforts were made to minimize discomfort, stress and pain to the animals. Sampling occurred following the lethal anesthesia of fish with an overdose of the same chemical.

### 4.2. Production of Antisera Raised against Sea Bass TRγ Peptides

Three peptides (NH2-YPAASRAHLEGKISC-COOH; NH2-KVTSADKKQLKLKESGC-COOH; and NH2-LMIFRNKGPSTNCTH-COOH) were synthetically produced based on the T-cell receptor gamma chain protein sequence (NCBI GenBank nucleotide and protein accession #FR745889.1 and #CBY79008) (Primm Srl, Milano, Italy) and used as antigens for anti-peptide antisera production. Two New Zealand rabbits were immunized subcutaneously with a mix of the three keyhole limpet hemocyanin (KLH)-conjugated peptides resuspended in 0.1 M PBS and added with complete Freund’s adjuvant (Serva, Heidelberg, Germany) in the first two inocula. The obtained PAbs were named RaTRγ1 and RaTRγ2. 

### 4.3. RNA Extraction and RT-PCR

Total RNA was extracted from 3 pools of specimens sampled at different dph (0, 3, 7, 10, 14, 17, 21, 24, 28, 31, 35, 37, 42, 45, 49, 52, 56 and 63) (*n* = 30) and from the thymus and intestine of post-larvae at 73, 88, 93 dph and 1-year-old specimens (*n* = 3) fed with rotifers, artemia and a commercial diet according to age. Samples were ground with a sterile pestle in 1 ml of TRIsure (Bioline, catalog #BIO-38032) and extracted according to the manufacturer’s instructions. RNA was resuspended in diethyl-pyrocarbonate-treated water (DEPC; Merck, Germany). Two μg of total RNA were reverse transcribed with the BioScript™ MMLV Reverse Transcriptase (Bioline, catalog #BIO-27036) and RT-PCRs were performed using the Premix Taq™ Hot Start Version (Takara Bio, catalog #R028A) and selected primer pairs (Table 1). PCR products were purified from agarose gel using the QIAquick gel extraction kit (QIAgen) and directly sequenced with the MWG sequencing services to confirm the identity of transcripts. *TRβ*-specific primers were previously used in Picchietti et al. [50] and the amplicon cloned. The lack of DNA contamination was assessed by amplifying the cDNA with *β-actin* primers that bracketed an intron. The cycling protocol was: 5 min at 94 °C; 30 cycles of 45 s at 94 °C, 45 s annealing at primer specific temperature and 45 s at 72 °C; then 10 min at 72 °C.

### 4.4. Basal Expression Analysis of TRγ Transcripts in One-Year-Old Fish

Total RNA was isolated from the spleen, PBL, brain, liver, gut, thymus, HK, gills and muscle of one-year-old specimens fed with a commercial diet (*n* = 3) with TRIsure. RNA was resuspended in DEPC-treated water and used for real-time qPCR without pooling biological replicates. All samples were verified for purity and concentration on a spectrophotomer (Picodrop Ltd.) and for integrity of 28S and 18S rRNA on a 1% agarose gel stained with GelRed nucleic acid stain. Two μg of total RNA were reverse transcribed with BioScript™ MMLV Reverse Transcriptase. DNA contamination was assessed as per the previous section while *TRγ* transcript was obtained with specific primers (Table 1), which amplified a 200 bp product. The relative expression levels were determined with a Mx3000P qPCR System (Agilent Technologies, Inc., Wilmington, DE, USA) using 18S rRNA as the housekeeping gene and muscle as a calibrator (i.e., the tissue with the lowest TRγ expression) with the Pfaffl’s efficiency-calibrated method [73]. Results were calculated by averaging the expression values from three biological replicates, each analyzed in duplicate, and expressed as mean + SD.

### 4.5. Immunoprecipitation of Head Kidney Leukocyte Lysate with RaTRγ1 IgG

HK leukocytes were obtained from juvenile specimens fed with a commercial diet (*n* = 3) by teasing of the tissue with 100 and 40 µm nylon mesh and subsequent centrifugation through a Percoll density gradient (Amersham Pharmacia). Leukocytes were washed with PBS and lysed in 1 ml milliQ water containing PMSF and 10 mM CHAPS. The lysate was subjected to 10 freeze-thaw cycles, homogenized with a 1 ml Dounce homogenizer and centrifuged at 18,000 rcf (relative centrifugal force) for 5 min. The supernatant was incubated O/N with 10% (*v*/*v*) PAb RaTRγ1 IgG at 4°C in a 1.25 ml total volume under continuous mixing. As negative control, PBS was used instead of the protein lysate. The mixtures were then added to 30% (*v*/*v*) Protein G Sepharose^®^ slurry for IgG binding and incubated at 4 °C for 4 hours; subsequently, solutions were centrifuged three times at 3000 rcf for 5 min. The two resulting supernatants (i.e., the lysate fraction not bound by RaTRγ1 IgG or PBS alone) and pellets (i.e., the lysate fraction bound by RaTRγ1 IgG or RaTRγ1 IgG) were treated separately and each added to 50% (*v*/*v*) of 2x Laemmli solution. The samples were heated at 100 °C for 1 min and resolved on a 12% SDS-PAGE stained with the ProteoSilver™ Silver Stain Kit (Merck, catalog #PROTSIL1). The gel was visualized with the ChemiDoc™ imager, model XRS+ (Bio-Rad), from which an image file was exported. The image was corrected for brightness, contrast and color tone with Adobe Photoshop CC.

### 4.6. Flow Cytometry

Following dissection, lymphoid organs (thymus, HK and spleen), mucosal tissues (gills and gut) and PBL (*n* = 3) were maintained in cold HBSS on ice until teasing through 100 and 40 µm nylon mesh (except for PBL). A minimum of 5 × 10^6^ leukocytes from each tissue were obtained following an established protocol [74], with modifications for the gut tissue as reported in [75]. For the IIF assay, leukocytes were fixed with 0.4% PFA in PBS for 15 min on ice, washed in PBS by centrifugation at 500 rcf for 10 min and incubated for 1 h at 4 °C with either pre-immune serum or RaTRγ1 IgG at a 1:20 dilution in PBS. Subsequently, cells were washed with PBS at 500 rcf for 10 min and incubated for 45 min with a 1:200 dilution of an anti-rabbit IgG secondary antibody conjugated with CF^®^ 488A (Merck, catalog #SAB4600044). Negative controls were incubated with the secondary conjugates only. Fluorescence was detected with a Beckman–Coulter Epics XL-MCL flow cytometer and analyzed by acquiring 10,000 events with the Expo32 system software. The same software was used for overlaying the FL1 log histograms of pre-immune and RaTRγ1 IgG^+^ samples and highlighting gated events in the FS/SS dot-plots.

All media and solutions used for this analysis were brought to sea bass osmolarity (350 mOsm/kg) using 2M NaCl.

### 4.7. Immunohistochemical Investigation on Post-Larvae and One-Year-Old Fish, and Immunofluorescence Analysis

Whole post-larvae (*n* = 3) at 76 and 93 dph and thymus, spleen, HK, gut and gills from 1-year-old fish fed with a commercial diet (*n* = 3) were dissected and fixed in ice-cold Bouin’s fixative for 7 h at 4 °C, then dehydrated, embedded in paraffin wax and cut into 7-μm-thick sections using a rotary microtome. After dewaxing in toluene and rehydration in graded ethanol, IHC was performed by the ABC-peroxidase method. Briefly, serial sections were incubated for 18 h at room temperature with RaTRγ1 IgG diluted 1:100 in PBS containing 5% normal goat serum and 0.1% sodium azide. As NC, the PAb was replaced by pre-immune serum. Sections were incubated for 60 min with biotinylated goat anti-rabbit IgG serum (Vector Labs., Burlingame, CA, USA) diluted 1:1000 in PBS containing 0.1% sodium azide and 1% BSA, followed by incubation for 60 min with avidin biotinylated peroxidase complex (ABC, Vectastain Elite, Vector). Following rinses and staining with 3,3′-Diaminobenzidine (DAB) tetrahydrochloride liquid substrate system (Sigma), sections were dehydrated, mounted and examined under bright-field illumination. In each specimen, ten sets of five serial sections were processed for immunohistochemistry and five serial sections were stained with May–Grünwald/Giemsa (MGG) for general histology. 

For IIF, PI from one-year-old specimens (*n* = 3) were fixed in 4% PFA in 0.1M PBS, pH 7.4, O/N. Tissues were transferred to 25% sucrose in PBS, at 4 °C for 12 h, and then cut into 15 μm thick sections with a cryostat. Serial sections were mounted onto positively charged slides and stored at −20 °C until use. Sections were treated for 60 min with 5% bovine serum albumin BSA and 10% normal goat serum (Thermo Fisher Scientific, Sacramento, CA, USA) in PBS containing 0.5% Triton X-100. Incubation was performed with RaTRγ1 IgG primary antibody diluted 1:100 as above at 4 °C O/N. Following rinses in PBS, sections were incubated for 90 min with the Alexa Fluor 594 secondary antibody (Thermo Fisher Scientific) and finally cover-slipped with Fluoroshield Mounting Medium containing DAPI.

Images were acquired by a Zeiss Axioskop 2 plus a microscope equipped with the Axiocam MRC camera and the Axiovision software (Carl Zeiss, Oberkochen, Germany). Images were optimized for contrast and brightness using Adobe Photoshop CC.

### 4.8. Pre-Embedding Labeling of Cell Surface Antigens for Electron Microscopy

Intestinal leukocytes from three one-year-old specimens fed with a commercial diet were isolated as above and prefixed with 2% PFA in PBS, pH 7.4, for 10 min at RT. Samples were then washed three times in PBS (pH 7.4) containing 50 mM glycine to quench aldehydes. A block step was made for 30 min in 1% BSA and 10% NGS (British Biocell International) in PBS (pH 7.4), followed by washings with 1% BSA in PBS. Cells were incubated with PAb RaTRγ1 IgG as primary antibody at a dilution of 1:50 in 1% NGS, 0.1% Tween 20, 1% BSA and 0.1% sodium azide in PBS, pH 8.2 (buffer A), for 1 h in a moist chamber. The primary antibody was omitted in the negative control. Cells were then washed twice in buffer A and incubated for 1 h in a moist chamber with a secondary gold-labelled goat anti-rabbit antibody (gold particles of 10 nm diameter) (British Biocell International), diluted 1:10 in buffer A. After rinsing twice with PBS (pH 7.4), cells were fixed in 1% glutaraldehyde in PBS for 20 min at 4 °C and washed twice in distilled H_2_O. Samples were then post-fixed in 0.5% osmium tetroxide (Agar Scientific) in distilled H_2_O for 30 min and washed twice in distilled H_2_O. Cells were dehydrated with a graded ethanol series and embedded in medium grade LR White resin (Agar Scientific) for 48 h at 50 °C. Tightly capped gelatine capsules were used to allow resin polymerization. Ultrathin sections were obtained using a Reichert Ultracut ultramicrotome with a diamond knife and collected on nickel grids. Sections were subsequently stained with uranyl acetate and lead citrate and observed with a Jeol JEM EX II transmission electron microscope at 100 kV.

### 4.9. Diet Trials

Four isoproteic (46.3% crude protein as fed basis) and isolipidic (17.5% crude lipids as fed basis) experimental diets were formulated. A PC diet was formulated with 30% fish meal and fish oil as the only lipid source. A NC diet based on plant ingredients and low fishmeal (15%) level was also formulated, including a mixture of fish and vegetable oil (40/60) as a lipid source. Tested diets were formulated similarly to the NC, replacing 50 and 100% fish meal with *Nannochloropsis* sp. biomass, and named NANNO 7.5 and NANNO 15, respectively. The microalgae biomass was produced in closed production systems (photo bioreactor) and lyophilized by Buggypower Lda (Porto Santo, Portugal). Experimental diets were produced by extrusion by SPAROS (Olhão, Portugal), and each diet was tested in duplicate.

Sea bass juveniles of 24 ± 1 g average body weight (BW) were obtained from the commercial hatchery in Acuinuga (Spain) and transported to the experimental facilities to be quarantined for approximately 3 weeks. During this period, fish were fed a commercial diet suitable for sea bass juveniles (AquaGold, Aquasoja; Sorgal, S.A., São João de Ovar, Portugal). Twenty-five fish were randomly assigned to 8 homogeneous experimental groups receiving four diet formulations in duplicate. They were hand-fed twice a day, 6 days a week, until apparent visual satiation, for 11 weeks. Utmost care was taken to ensure that all feed supplied was consumed. The diets trial was conducted in a thermo-regulated recirculating water system, equipped with 8 square-shaped fiberglass 100 L tanks, supplied with a continuous flow of filtered seawater aerated by diffusion through air stones. During the trial, water temperature averaged 23.0 ± 0.5 °C, salinity was maintained at 35.0 ± 1.0 g L^−1^, and dissolved oxygen was kept near saturation (7.0 mg L^−1^). The photoperiod regime adopted was 12:12 hours light:dark, provided by artificial illumination. Three fish per tank, hence 6 fish per experimental group, were randomly collected. For IHC analysis the mid intestine (MI; considered the portion located mid length from the pyloric caeca to the anus) and posterior intestine (PI; distinguishable by an enlarged diameter and darker mucosa) were dissected and processed for IHC as described above (see Section 4.7).

### 4.10. Statistics

For the diet experiment, multiple sets of consecutive sections were counted for immunoreactive cells (nucleated only) in 100,000 µm^2^ areas (from fifteen non-consecutive sections per specimen) by an observer unaware of treatments. Cell measurements were obtained using a computer-assisted image analysis system which included a Zeiss microscope equipped with a color video camera (AxioCamMRC, Arese, Milano, Italy) and a software package (AxioVision). Results were expressed as the mean ± SD and statistical analysis was performed using a two-way ANOVA followed by Bonferroni’s multiple comparison test (selected pairs).

Real-time qPCR data were expressed as mean + SD and statistically analyzed with a one-way ANOVA followed by a Tukey post hoc multiple comparison test after having verified the fulfilment of parametric conditions.

In both cases, data analysis was performed using the Graph Pad Prism software, v.9, and statistical significance was set at *p* < 0.05.

## Figures and Tables

**Table 1:** Primer sequences used for RT- and q-PCR analysis.

Gene	Primers Sequence 5′-3′ (Forward, FW, and Reverse, RV)	Accession Number
*β-actin*	ATGTACGTTGCCATCC (FW, RT) GAGATGCCACGCTCTC (RV, RT)	AJ493428
*18S rRNA*	CCAACGAGCTGCTGACC (FW, q) CCGTTACCCGTGGTCC (RV, q)	AY831388
*TRγ*	CTGCTGTGTGTGGCCTCAGAC (FW, RT) GTGCTGGACGGAGCAGTGGTA (RV, RT) CTGCTGTGTGTGGCCTCAGAC (FW, q) AGCAAGAGAGTCCACAGCAGT (RV, q)	FR745889
*TR* *β*	AGATTACCGGACCATCAGTGAAAG (FW, RT) TCAGTAGTTCTGCTTTCCCTTTGA (RV, RT)	AJ493441
*CD3* *ε*	CCTTACCACTGTAAATATGAGGACG (FW, RT) CAGGTTGACTCCGGGCTGCTG (RV, RT)	KX231274

## Data Availability

Data available upon request.

## Supplementary Materials

Supplementary materials can be found at https://www.mdpi.com/1422-0067/22/7/3376/s1. Figure S1: ELISA test against a mix of peptides conjugated to OVA using preimmune and immune sera from rabbit 1 and 2. Based on higher O.D. values obtained with immune serum from rabbit 1, this was selected and IgG-purified for all subsequent analysis, and named RaTRγ1 IgG.

## Abbreviations

AI	anterior intestine
ANOVA	analysis of variance
BSA	bovine serum albumin
BW	body weight
CHAPS	3-((3-cholamidopropyl) dimethylammonio)-1-propanesulfonate
DAPI	4′,6-diamidino-2-phenylindole
DP	double positive
dph	day post hatching
DN	double negative
E	epithelium
HBSS	Hank’s Balanced Salt Solution without calcium and magnesium
HK	head kidney
kDa	kilo Dalton
IIF	indirect immunofluorescence
LP	lamina propria
L	lumen
MHC	major histocompatibility complex
MI	mid intestine
MW	molecular weight
MMCs	melano-macrophage centres
NC	negative control
NGS	normal goat serum
O/N	overnight
PAb	polyclonal antibody
PBL	peripheral blood lymphocytes
PBS	phosphate-buffered saline
PBT	PBS, Triton and BSA
PC	positive control
PFA	paraformaldehyde
PI	posterior intestine
PMSF	phenylmethylsulfonyl fluoride
RT	room temperature
TcR	T cell receptor
TR	T cell receptor chain
